# Evaluation of the Antibacterial Activity of Crude Extracts Obtained From Cultivation of Native Endophytic Fungi Belonging to a Tropical Montane Rainforest in Colombia

**DOI:** 10.3389/fmicb.2021.716523

**Published:** 2021-09-17

**Authors:** Esteban Charria-Girón, María C. Espinosa, Andrea Zapata-Montoya, María J. Méndez, Juan P. Caicedo, Andrés F. Dávalos, Beatriz E. Ferro, Aida M. Vasco-Palacios, Nelson H. Caicedo

**Affiliations:** ^1^Departamento de Ingeniería Bioquímica, Facultad de Ingeniería, Universidad Icesi, Cali, Colombia; ^2^Departamento de Ciencias Biológicas, Facultad de Ciencias Naturales, Universidad Icesi, Cali, Colombia; ^3^Departamento de Salud Pública y Medicina Comunitaria, Facultad de Ciencias de la Salud, Universidad Icesi, Cali, Colombia; ^4^Grupo de Microbiología Ambiental - BioMicro, Escuela de Microbiología, Universidad de Antioquia (UdeA), Medellín, Colombia; ^5^Asociación Colombiana de Micología (ASCOLMIC), Medellin, Colombia; ^6^Centro BioInc, Universidad Icesi, Cali, Colombia

**Keywords:** antibacterial activity, fungal endophytes, secondary metabolites, co-culturing strategies, *Rainforest ecosystem*

## Abstract

Bioactive secondary metabolite production from endophytic fungi has gained a recurring research focus in recent decades as these microorganisms represent an unexplored biological niche for their diverse biotechnological potential. Despite this focus, studies involving tropical endophytes remain scarce, particularly those isolated from medicinal plants of these ecosystems. In addition, the state of the art of the pharmaceutical industry has experienced stagnation in the past 30years, which has pushed pathogenic infections to get one step ahead, resulting in the development of resistance to existing treatments. Here, five fungal endophytes were isolated from the medicinal plant *Otoba gracilipes* (Myristicaceae), which corresponded to the genera *Xylaria* and *Diaporthe*, and screened to demonstrate the promissory potential of these microorganisms for producing bioactive secondary metabolites with broad-spectrum antibacterial activities. Thus, the evaluation of crude organic extracts obtained from the mycelia and exhaust medium allowed the elucidation of *Xylaria* sp. and *Diaporthe endophytica* potential toward providing crude extracellular extracts with promising bioactivities against reference strains of *Escherichia coli* (ATCC 25922) and *Staphylococcus aureus* (ATCC 25923), according to the determined half-maximum inhibitory concentration (IC_50_) with values down to 3.91 and 10.50mg/ml against each pathogen, respectively. Follow-up studies provided insights into the polarity nature of bioactive compounds in the crude extracts through bioactivity guided fractionation using a polymeric resin absorbent alternative extraction procedure. In addition, evaluation of the co-culturing methods demonstrated how this strategy can enhance endophytes biosynthetic capacity and improve their antibacterial potential with a 10-fold decrease in the IC_50_ values against both pathogens compared to the obtained values in the preliminary evaluations of *Xylaria* sp. and *D. endophytica* crude extracts. These results support the potential of Colombian native biodiversity to provide new approaches concerning the global emergence of antibiotics resistance and future production of undiscovered compounds different from the currently used antibiotics classes and simultaneously call for the value of preserving native habitats due to their promising ecosystemic applications in the biotechnological and pharmaceutical industries.

## Introduction

The current health crisis that has arisen from the spread of antimicrobial resistance has become one of the major causes of death occurring worldwide, accounting for more than 700,000 deaths annually, which in turn is threatening the global community development and modern medical achievements (World Health Organization, 2019). The abovementioned points expose the pharmaceutical industry’s failure to provide effective and innovative treatments to defeat multidrug-resistant (MDR) pathogens, which are increasingly becoming prevalent worldwide (Nikaido, 2009; Brown and Wright, 2016). Although the conventional approaches have proven to be effective during the last century, the most recently developed drugs are not effective against several bacteria’s evolving resistance systems. This situation has resulted from the diverse mechanisms of antimicrobial resistance, which rely on the following principles: the synthesis of hydrolytic enzymes, the modification of active binding sites, decreasing antimicrobial affinity, decreasing cell-wall permeability to drugs, and the generation of efflux pumps, which transport the antimicrobial agents outside cells (Reygaert, 2018). Thus, the lack of new and effective antimicrobial agents shows the urgent need for novel sources of bioactive compounds beyond the conventional antibiotic classes (Ruddaraju et al., 2020). Historically, most antibiotics belong to a limited group of molecular scaffolds whose lifespan has been extended through several generations of synthetic modifications (Ogawara, 2021). In this way, most of the main classes of antibiotics have been discovered through systematic bioprospection of soil microbes, which led them to advance in synthetic chemistry. However, the recent advances in mycology have unearthed the biosynthetic diversity of fungi toward providing novel antibiotics in response to the new millennium crisis.

In this sense, fungal endophytes, which can survive inside plant tissues for brief or prolonged periods without producing any visible symptoms, are a promising feedstock for future antimicrobials (Gupta et al., 2020). This fact can be attributed to their extraordinary ecological relationship with the host plants, which has led to discovering novel compounds (Rambold et al., 2013; Helaly et al., 2018; Sandargo et al., 2019). This ecological interaction has provided endophytes with versatile biosynthetic pathways capable of producing previously undiscovered secondary metabolites with potentially beneficial properties and applications, including antimicrobials, antivirals, antifungals, anticarcinogens, immunosuppressants, and antioxidants (Rambold et al., 2013). In the last decade, the Ecology-Bioprospecting-Bioprocessing (EBB) research group from Universidad Icesi has focused on bioprospecting rainforests in the Valle del Cauca, Colombia, for the promotion of both the value of biodiversity and the native ecosystemic services. Recently, the EBB-research group has isolated more than 30 fungal endophytic species from *Otoba gracilipes* (Family: Myristicaceae; Common name: Otobo) with various potential applications. We explored the secondary metabolites produced by fungal endophytes of *O. gracilipes*, a tropical medicinal tree associated with a montane rainforest ecosystem, poorly explored for potential bioactive metabolites (Caicedo et al., 2019). A particular type of ecosystem that is supposed to possess an unearthed capacity to produce novel compounds in response to survival adaptation strategies acquired under the adverse conditions in these ecosystems are scarce (Sousa et al., 2016; Martinez-Klimova et al., 2017). On the other hand, a recent report indicates that *O. gracilipes* is one of seven threatened forest species in the Valley of Cauca river geographic area (Bonilla, 2020). Therefore, it is essential to continue studying the endophytic fungal diversity and its biotechnological potential on this species to preserve the value of these endangered ecosystems.

Some isolated strains belonging to the genera *Xylaria* (Xylariaceae, Xylariales, and Ascomycota) and *Diaporthe* (Diaporthaceae, Diaporthales, and Ascomycota) have been identified among the *O. gracilipes* endophytes, which have provided novel bioactive molecules in the past decade. The *Xylaria* and *Diaporthe* generas represent one of the scarcest sources of unidentified and promising secondary metabolites (Sousa et al., 2016; Helaly et al., 2018). Recent studies have demonstrated the biosynthetic capacity of *Xylaria*, providing a new source of bioactive molecules, including sesquiterpenoids, diterpenoids, diterpene glycosides, triterpene glycosides, steroids, organic nitrogenous compounds, and aromatic compounds as well as the derivatives of pyrones and polyketides (Song et al., 2014). Most of these molecules display antibacterial, antifungal, phytotoxic, anticancer, cytotoxic, and anti-inflammatory activities. Similarly, some species of *Diaporthe* can produce unique natural products with low molecular weights and a variety of bioactivities, including antibacterial, anticancer, antifungal, antiviral, cytotoxic, and herbicide activities. Specifically, some endophytic strains of *Xylaria* generate compounds with antibacterial activities, such as the polyketide, mycoalexine, 3-O-methylmethylmellein, nortriterpenoid Helvolic acid (Ratnaweera et al., 2014), and the cyclopentapeptide Xylapeptide A (Xu et al., 2017); similarly, an endophytic strain of *Diaporthe* sp. produces Diaporone A, a new antibacterial secondary metabolite (Guo et al., 2020). Also, strains as *Phomopsis* sp. and *Phomopsis longicolla* S1B4 produce phomoneamide and dicerandrol A-C, respectively, which are the compounds with promissory antibacterial activity (Chepkirui and Stadler, 2017; Becker and Stadler, 2020).

On the other hand, it is well-known that, in natural ecosystems, interspecies relationship plays an important role in fungi’s behavior, particularly to fungal endophytes, which interact continuously with other endophytic microorganisms and their host plants (Mani et al., 2015). Surprisingly, these interactions can lead to several biochemical changes within their metabolism, such as the regulation of silent gene clusters related to the biosynthesis of the secondary metabolite, which is presumed to be silenced under the laboratory axenic culture conditions (Brakhage et al., 2008; Deepika et al., 2016). A recent revision of strategies such as co-cultures has demonstrated its promissory potential to replicate the ecological conditions by mimicking the endophytic communities (Chagas et al., 2013). These studies have demonstrated a capacity to provide an effective platform for discovering novel compounds with diverse chemical nature and industrial applications. This hypothesis has been too evaluated with macrofungi (basidiomycetes) to investigate the expression of silent genes in symbiotic systems linked with metabolomics study. One hundred thirty-six fungi-fungi symbiotic systems were built up by co-culturing 17 strains, among which the co-culture of *Trametes versicolor* and *Ganoderma applanatum* demonstrated the strongest colouration of confrontation zones; discovering that 62 features were either newly synthesized or highly produced in this co-culture (Yao et al., 2016). Similarly, several past studies have reported that metabolites secreted during monoculture are promoted when cultured in combination with other microorganisms, increasing bioactive metabolite’s productivities (Zhu et al., 2018).

In this study, the antimicrobial potential of five native fungal endophytes isolated from medicinal plant of the Colombian rainforest was assessed by evaluating their crude organic extracts against antibiotic-susceptible pathogens *Escherichia coli* (ATCC 25922, Gram-negative) and *Staphylococcus aureus* (ATCC 25923, Gram-positive). These strains were preselected from all the isolates due to they belong to well-recognized genus with ability to synthetize secondary metabolites with antimicrobial activity. Furthermore, the development of a co-culture platform between *Diaporthe endophytica* and *Xylaria* sp. was systematically investigated, where the antagonist interaction between them improved the crude extract effectivity. Similarly, an alternative extraction with a polymeric resin absorbent was used to enhance the selective capture of bioactive compounds according to their molecular weight and polarity, gaining valuable insights for future purification efforts. The presented study also emphasizes on the crucial role of systematical screening platforms in the rapid and effective prioritization of antimicrobial agents prior to chemical investigation of bioactive molecules. In some cases, the cost and investing time associated to the identification of new antimicrobial compounds could be avoid by the screening of microorganisms, which are previously known to produce specific potential antimicrobial agents into crude extracts (Caicedo et al., 2011; Santiago et al., 2021). Nonetheless, our results provide an initial step toward bioprospecting the Colombian southwestern endophyte diversity and highlight the value of preserving the native habitats owing to their promising ecosystemic applications in the biotechnological and pharmaceutical industries.

## Materials and Methods

### Study Area and Collection of Plant Materials

Fresh and healthy leaves and stems of two young trees of *O. gracilipes* were collected during the dry season (November, 2019) in the Natural Reserve “La Carolina” (3°24'10.662''N, 76°36'52.774''W), Cali, Valle del Cauca, Colombia at 1,600m.a.s.l. The plant material was collected and cut with a sterile scalpel and stored at 4°C in a sterile polyethylene bag until further use.

### Endophytic Fungi Isolation

The processed material was surface-sterilized by washing thoroughly in sterile demineralized water, followed by that with 70% ethanol for 1–2min and 3% sodium hypochlorite for 15min (Caicedo et al., 2019). Small pieces of plant tissues were then placed on potato dextrose agar (PDA, Merck®, Darmstadt, Germany) medium at pH 6.0, which was supplemented with clindamycin (0.2ml/100ml) in Petri dishes and incubated at 29°C until the fungus started to grow (Prihantini and Tachibana, 2017). Pure isolates with distinct morphology were selected for further molecular identification. Each fungal strain was preserved on PDA with mineral oil and sub-cultured in the same solid media before performing genomic DNA extraction and fermentation experiments.

### Molecular Identification of Endophytic Fungi

Molecular identification was conducted using the previously grown strains on PDA and incubated for 5–7days at 29°C. The fungal DNA was extracted using the EZNA® Tissue DNA Kit (Omega Bio-Tek, Norcross, GA, United States), and the complete DNA profile was quantified (>100ng/μl for a volume of 25μl) using the NanoDrop Spectrophotometer 2000/2000c ND-1000 (NanoDrop, Wilmington, DE, United States). The reaction mixture contained: buffer PCR, DNTPs, MgCl_2_, TAQ Pol, Primer F, and Primer R. The nuclear ribosomal ITS1 region was amplified with the primers ITS1 and ITS5 (White et al., 1990). DNA amplification was performed in the Swift – MiniPro Thermal Cycler (ESCO, Singapore) with an initial denaturation step for 1min at 95°C, followed by 35cycles of denaturation for 1min at 95°C, annealing for 30s at 52°C, and an extension for 30s at 72°C. A final extension was performed at 72°C for 5min. The PCR products were visualized using 1% agarose gel. Purification of the products was conducted using the Wizard S.V. Gel and PCR Clean-Up System (Promega, San Luis Obispo, CA, United States) before being subjected to sequencing protocols using the Applied Biosystems® ABI Prism 3,500 Sequencers (Thermo Fisher Scientific, Waltham, MA, United States). The resulting DNA sequences were analyzed and compared with those obtained from the GenBank *via* a BLAST search. The sequences from our study were also deposited in GenBank.

All the protocols and procedures employed in this investigation were verified and approved by the appropriate institutional review committee. The specimens were kept and handled in accordance with the guidelines of the National Environmental Licensing Authority (ANLA) of Colombia, through the Framework Permit for the Collection of Specimens of Wild Species of Biological Diversity for Non-Commercial Scientific Research Purposes – Resolution 0526, May 20, 2016. Furthermore, according to the Resolution 0364, March 12, 2018, and addendum to contract No. 4 of the Framework Contract for Access to Genetic Resources and their Derivative Products No. 180 of 2018, Universidad Icesi has the acceptance to the request of Framework Contract for Access to Genetic Resources and their Derivative Products for the Program for the Study, Use, and Sustainable Use of Colombian Biodiversity.

### Fermentation of Fungal Isolates

For the primary screening, endophytic fungi were cultured in 250-ml Erlenmeyer flasks containing 150ml of potato dextrose broth (PDB) by triplicate for all five isolated strains (Rao et al., 2015). Four agar plugs of 5-mm diameter from a 7-day PDA plate were used as inoculum for each experimental unit. Flask fermentations were incubated at 29°C (pH 6.0) under orbital agitation of 90rpm until carbon sources were depleted, according to the DNS assay for reducing sugars (Ghose, 1987). After the end of the culture period, the mycelium was separated from the fermentation broth through vacuum filtration using qualitative paper filters (0.45μm) for further extraction procedures.

### Extraction Procedures

#### Mycelial Organic Crude Extract

**Figure 1:**
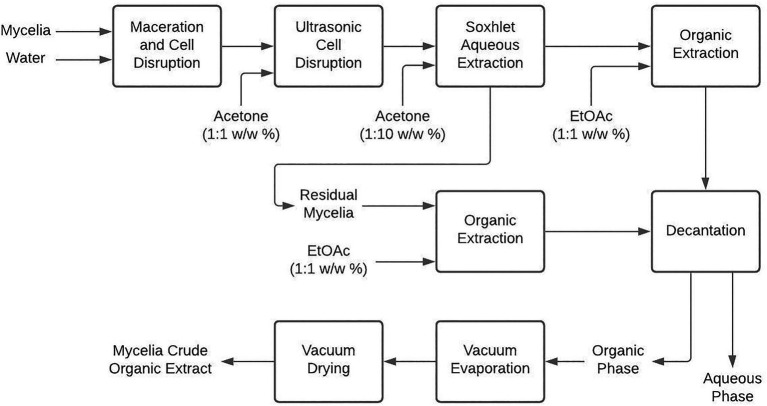
Mycelium extraction procedure to obtain organic crude extracts from liquid cultivation of endophytic fungi.

Once all the mycelia were obtained, they were subjected to maceration and breaking with glass spheres, followed by ultrasonic bath digestion with acetone (1:1 w/w) for 1h (Surup et al., 2019) and a Soxhlet extraction with acetone (1:10 w/w) for 1h. The resulting solution was extracted thrice in a ratio of 1:1 w/w with ethyl acetate (EtOAc) in a separation funnel. In addition, the mycelium was also extracted with EtOAc (1:5 w/w). The resulting decanted organic-phases were combined, reduced by vacuum-evaporation (40°C, 40mbar) to 5ml, and then vacuum-dried to finally obtain the organic crude extracts (Figure 1).

#### Extracellular Organic Crude Extract

**Figure 2:**
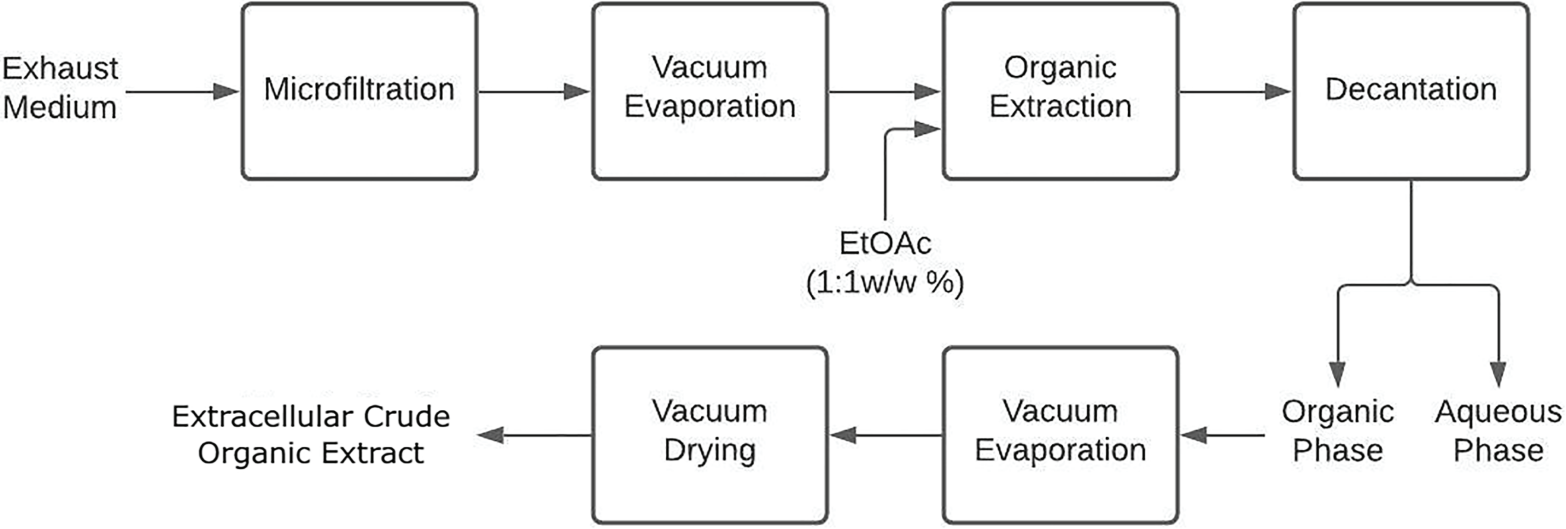
Extracellular extraction procedure to obtain organic crude extracts from liquid cultivation of endophytic fungi.

First, the exhausted culture medium was vacuum-filtered by gradually reducing the filter pore size (From 10 to 0.22-μm). The filtrate was then concentrated to 40ml by vacuum-evaporation (40°C, 20mbar) and extracted thrice with EtOAc (1:1 w/w) in a separation funnel. The organic phases followed the same procedure as in section Mycelial Organic Crude Extract to finally obtain extracellular crude extracts (ECEs) as depicted in Figure 2 (Surup et al., 2019).

### *In vitro* Antibacterial Assays

Two bacterial strains (Test organisms) were used in this study, including a Gram-positive bacteria *S. aureus* (ATCC 25923) and a Gram-negative bacteria *E. coli* (ATCC 25922). The bacterial cultures were reactivated in Luria Bertani broth medium (LB; Scharlau, Barcelona, Spain) at 37°C for 24h, followed by streaking on LB agar medium plate and incubating for 16h at 37°C before using in the assays.

#### Primary Screening for Antibacterial Activity

The antibacterial activity of organic crude extracts was evaluated against each test bacterium using a modified broth microdilution method (Caicedo et al., 2011) with four 2-fold dilutions starting from 25mg/ml of each extract dissolved in a dimethyl sulfoxide (DMSO) solution (1% v/v). Clear round-bottomed 96-microtiter plates (Brandtech®; Essex, Connecticut, United States) were set up with the extracts to be evaluated in triplicate using LB and the broth with DMSO (1% v/v) as a negative control. Each microbial suspension was previously adjusted to an optical density (OD 620nm) of 0.08–0.1, equivalent to 1×10^8^CFU/ml. Other dilutions were prepared with LB to yield 1×10^4^CFU/ml in the assays from this previous solution. The assay plates were then incubated at 37°C for 20h before estimating their OD_620_ using a microplate reader (Varioskan™ LUX Thermo Scientific™, United States).

For crude extracts prioritization, the normalized responses were calculated as described by Caicedo et al. (2012). A statistical cutoff of a 0.5 normalized response at 25mg/ml was selected in this order. Hence, the extracts that displayed a lower response than the cutoff value were scored as a positive hit. The half-maximum inhibitory concentration (IC_50_) was determined for each selected crude extract using the Drm package from R statistical software (Ranke, 2006). Hierarchical cluster analysis (HCA) was carried out using IC_50_ values determined above to identify influential groups regarding their bioactivity. For this purpose, *clustegram* function was implemented using MATLAB® 2019b (The MathWorks, Inc.).

### Extracellular Extraction Using Polymeric Resin Adsorbent

*Xylaria* sp. and *D. endophytica* were subjected to an alternative extracellular extraction procedure to obtain a more purified extract of each axenic culture grown on PDB. Hence, the mycelium was separated by filtration from the fermentation broth, after which the exhausted medium was extracted with polymeric resin Amberlite® XAD16N (Sigma-Aldrich, Darmstadt, Germany), previously washed with distilled water (1% w/v), methanol (1% w/v), and distilled water (0.5% w/v) again for 30min with each solution, respectively. This method was adapted from Caicedo et al. (2011) and Narmani et al. (2018). A ratio of 0.06g resin/ml of the exhausted media was applied and continuously mixed in an Erlenmeyer flask (100–500ml, depending on the supernatant volumes) using agitation plates (120rpm) at room temperature for 24h. For the extraction, 25ml of methanol (MeOH) and EtOAc per gram resin were added and continuously mixed for 4h. Then, the resin was filtered, and the organic solution was reduced by vacuum-evaporation (40°C, 40mbar) to 5ml, and then vacuum-dried to obtain the extracts finally in Caicedo et al. (2011). These last followed the same antibacterial activity assessment procedure, which was performed for the primary screening.

### Co-culturing Screening Platform

After the preliminary screening of antibacterial activity, *Xylaria* sp. and *D. endophytica,* endophytes prioritized by HCA, were selected to develop a further screening platform. In this case, first, an antagonism plate co-culture assessment between themselves and the other fungal evaluated strains was performed to identify the respective interactions through qualitative observation (Hamzah et al., 2018). Then, according to these results, co-culturing in liquid media was performed to obtain similar crude extracts for antibacterial assessments.

#### Antagonism Plate Assay

The antagonistic activities of the selected fungal strains were qualitatively assessed between the non-prioritized fungal strains and the two prioritized ones through a dual culture plate assay (Chagas et al., 2013). One agar plug of each strain was placed on the opposite sides of the plate, followed by incubation at 29°C for 7days on the solid medium of Yeast-Maltose-Glucose (YM; 10g/l malt extract, 4g/l yeast extract, and 4g/l D-glucose) previously adjusted to pH 6.3 (Shao et al., 2020).

#### Co-culture in Liquid Media

The dual culture assay between *Xylaria* sp. and *D. endophytica* was prioritized for its different liquid media studies. PDB (pH 6.0) and YM (pH 6.3) were evaluated for the selected co-culture system. Hence, each strain was individually pre-cultured in 150ml of this medium. After proper mycelial growth, the mycelium was harvested and washed twice with a sterile solution of 0.9% w/w NaCl before inoculation in 1-L flasks containing 500ml of each medium. Independent duplicates were incubated at 29±1°C under orbital agitation of 90rpm until all the carbon sources were depleted, according to the DNS assay for reducing sugars (Ghose, 1987). Then, the obtained ECEs were subjected to further evaluation of the antibacterial activity against the test bacteria.

### Statistical Analyses

Data processing and IC_50_ determination were performed using the R (R Core Team, 2020), and the dose-response curves were produced using the ggplot2 package. The HCA was realized using the MATLAB® numerical software.

## Results

### Molecular Identification of Endophytic Fungi

**Figure 3:**
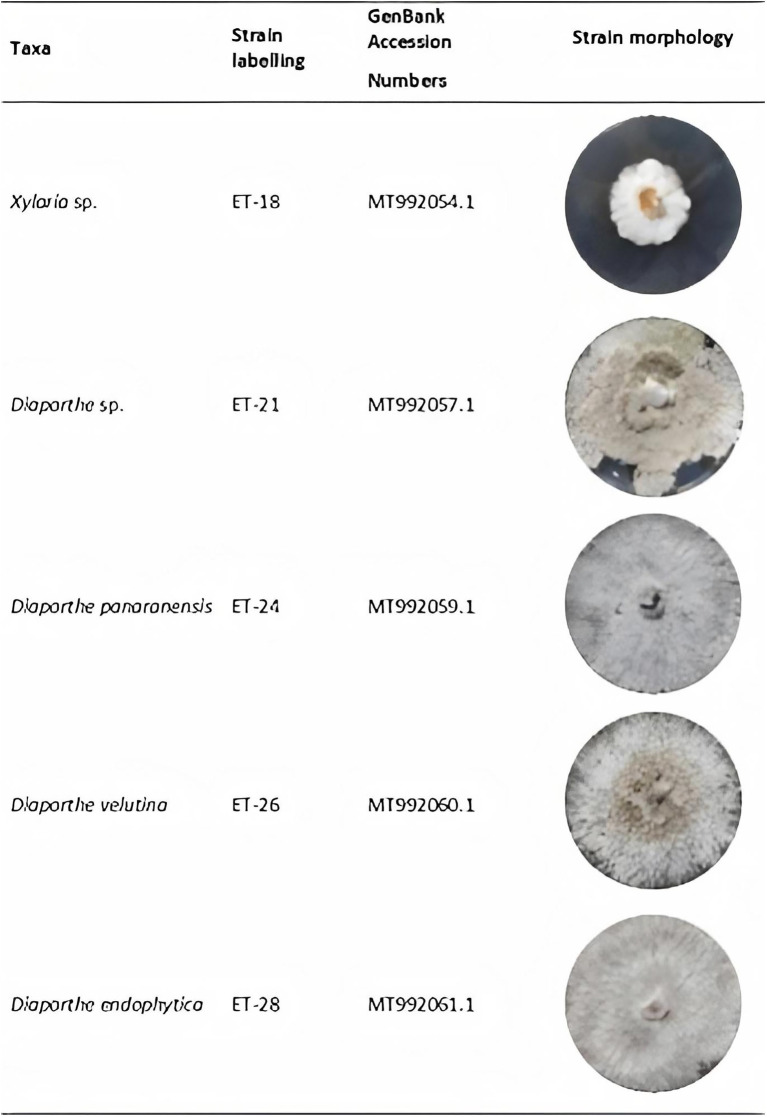
Taxonomic information of endophytic strains and mycelial growth on potato dextrose agar (PDA) after 8days incubation at 29°C.

Five strains of Ascomycota isolated as endophytes of *O. gracilipes* were obtained for this study (Figure 3). One strain belonged to the genera *Xylaria*, while the other four strains corresponded to *Diaporthe*, respectively. Each strain was identified at the species level through amplification, sequencing, and subsequent analysis using the rDNA’s ITS1 region. The sequences generated in this study were deposited in GenBank, and the associated accession numbers are indicated in Figure 3.

### Fermentation and Extraction

The production of crude metabolites from each fungal endophyte was performed for the primary screening in PDB. Among the cultured endophytes, *Diaporthe* strains displayed a faster growth than *Xylaria* sp. In addition, the *Diaporthe* strains could consume the available carbon sources rapidly. At the same time, *Xylaria* sp. showed a more extended adaptation phase, which corresponds to the low or non-initial consumption of glucose. However, according to the iodine-starch test, this carbon source was depleted faster than glucose, suggesting this endophyte’s carbon source predilection. On the other hand, *Diaporthe* endophytes consumed both the carbon sources simultaneously within a week in most cases.

**Figure 4:**
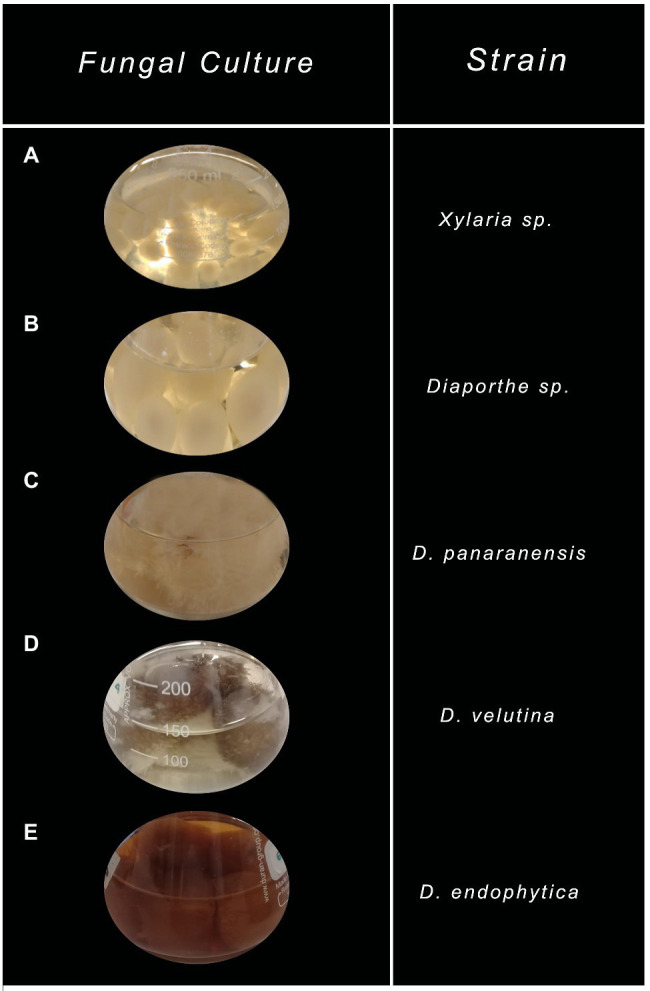
Characteristic of endophytic fungal cultures grown on potato dextrose broth (PDB) after depleting all carbon sources. **(A)** *Xylaria* sp., **(B)** *Diaporthe* sp., **(C)** *Diaphorte panaranensis.*, **(D)** *Diaporthe velutina*, and **(E)** *Diaporthe endophytica*.

On the other hand, morphological and coloration changes were observed in most of the ferementations. *Xylaria* sp. grew as pellets without coloring the fermentation broth compared to the control. In contrast, all *Diaporthe* endophytes grew as free mycelia and displayed various strain-depending colorations, where only *Diaphorte panaranensis* did not produce any coloration. *Diaporthe* sp. and *D. endophytica* exhibited a dark-brown and an orange to red colouration of the fermentation broth, respectively, in which *D. endophytica* produced colored compounds progressively as carbon sources were consumed. In the same way, *Diaphorte velutina* mycelia acquired a dark coloration and simultaneously clarified the broth, as depicted in Figure 4. Moreover, the mass of crude extract recovered from the exhaust medium was higher than that from the mycelial extracts. Nevertheless, the highest extraction yield of crude organic extract from the exhaust medium and the mycelium were obtained for *Xylaria* sp. (Table 1). Moreover, no significant differences (*p*>0.05) were noted between the extracellular extraction yields, contrary to the mycelial extraction values. Even between the *Diaporthe* strains, different values were obtained, as depicted in Table 1.

**Table 1:** Extraction yields of mycelial and ECEs from the cultivation of endophytic fungi isolated from *O. gracilipes*.

Strain	Mycelial crude extraction yield (mg crude extract/g mycelium)	Extracellular crude extraction yield (mg crude extract/g exhausted medium)
*Xylaria* sp.	9.3±1.2	13.0±1.8
*Diaporthe* sp.	1.5±1.2	12.9±1.6
*Diaporthe panaranensis*	8.4±1.3	11.3±2.5
*Diaporthe velutina*	1.82±1.1	11.7±2.8
*Diaporthe endophytica*	0.2±0.1	12.8±1.6

Each value represents the average of three independent biological replicates (*n*=3).

### Antibacterial Activity Screening

The antibacterial activities of crude organic extracts obtained from the exhaust medium and the mycelium were assessed against the antibiotic-susceptible bacteria *E. coli* (ATCC 25922) and *S. aureus* (ATCC 25923), which, according to the preliminary work, were more active than the crude metabolites obtained from aqueous extracts (data not shown). The results of antibacterial activity evaluations are summarized in Table 2. A reduced number of extracts were found to be effective against the gram-negative bacterium *E. coli*, with IC_50_ of 10.50 and 19.17mg/ml. In contrast, most of the evaluated extracts displayed moderate antibacterial activity against *S. aureus*. However, the extracellular and mycelial crude organic extract from *D. endophytica* exhibited promissory antibacterial activities, with IC_50_ values of 3.91 and 3.42mg/ml, respectively.

**Table 2:** IC_50_ for mycelial (MO) and extracellular (EO) crude organic extracts obtained from the cultivation of native fungal endophytes.

Extract	IC_50_ (mg/ml)	
	*Staphylococcus aureus*	*Escherichia coli*
1-MO	25	>25
1-EO	8.18	19.17
2-MO	13.66	>25
2-EO	>25	>25
3-MO	7.41	>25
3-EO	>25	>25
4-MO	3.25	>25
4-EO	>25	>25
5-MO	3.42	>25
5-EO	3.91	10.50

*Xylaria* sp. (1), *Diaporthe* sp. (2), *Diaphorte panaranensis* (3), *Diaphorte velutina* (4), and *Diaphorte endophytica* (5) against tested pathogens.

**Figure 5:**
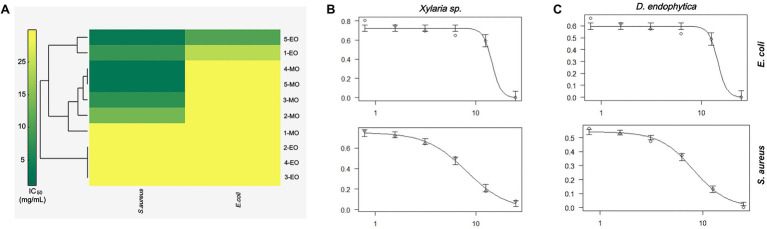
Antibacterial activity primary screening of endophytic strains of *Otoba gracilipes* **(A)** Hierarchical cluster analysis (HCA) of half-maximum inhibitory concentration (IC_50_) values for extracellular crude extracts (ECEs) obtained from the five fungal endophytes cultures against the evaluated pathogens. Dose-response curves were obtained for the ECEs of **(B)** *Xylaria* sp. and **(C)** *D. endophytica* against both pathogens, where the *y*-axis represents normalized response (0–1), while the *x*-axis represents extract concentration in mg/ml.

Hierarchical cluster analysis was later implemented to statistically identify groups with similar performance according to their IC_50_ values for each bacterium. As shown in Figure 5A, the ECEs from *Xylaria* sp. and *D. endophytica* were clustered under the same group. Both the extracts displayed bioactivity against both Gram-negative and Gram-positive bacteria. On the other side, the remaining extracts were clustered as extracts with antibacterial activities against *S. aureus* and those with no identified bioactivity against any bacteria under the evaluated range of concentrations. Remarkably, even when the mycelial organic extracts displayed a lower IC_50_ value than that of the ECEs, in most cases, no antibacterial activity was recorded against *E. coli* for the evaluated mycelial crude metabolites. In addition, to generate appropriate dose-response curves for the respective ECEs of the prioritized strains, a new evaluation was realized with two additional dilutions (25–0.78125mg/ml; Figures 5B,C).

### Extracellular Extraction Using Polymeric Resin Adsorbent

According to the primary screening, the ECEs from *Xylaria* sp. and *D. endophytica* exhibited the highest antibacterial activity. For this reason, an alternative procedure was used to recover extracellular organic metabolites with the polymeric resin Amberlite XAD-16. This resin is a non-ionic, hydrophobic, cross-linked polymer with a macro reticular structure and a high surface area commonly used to select organic molecules of relatively low molecular weight.

**Figure 6:**
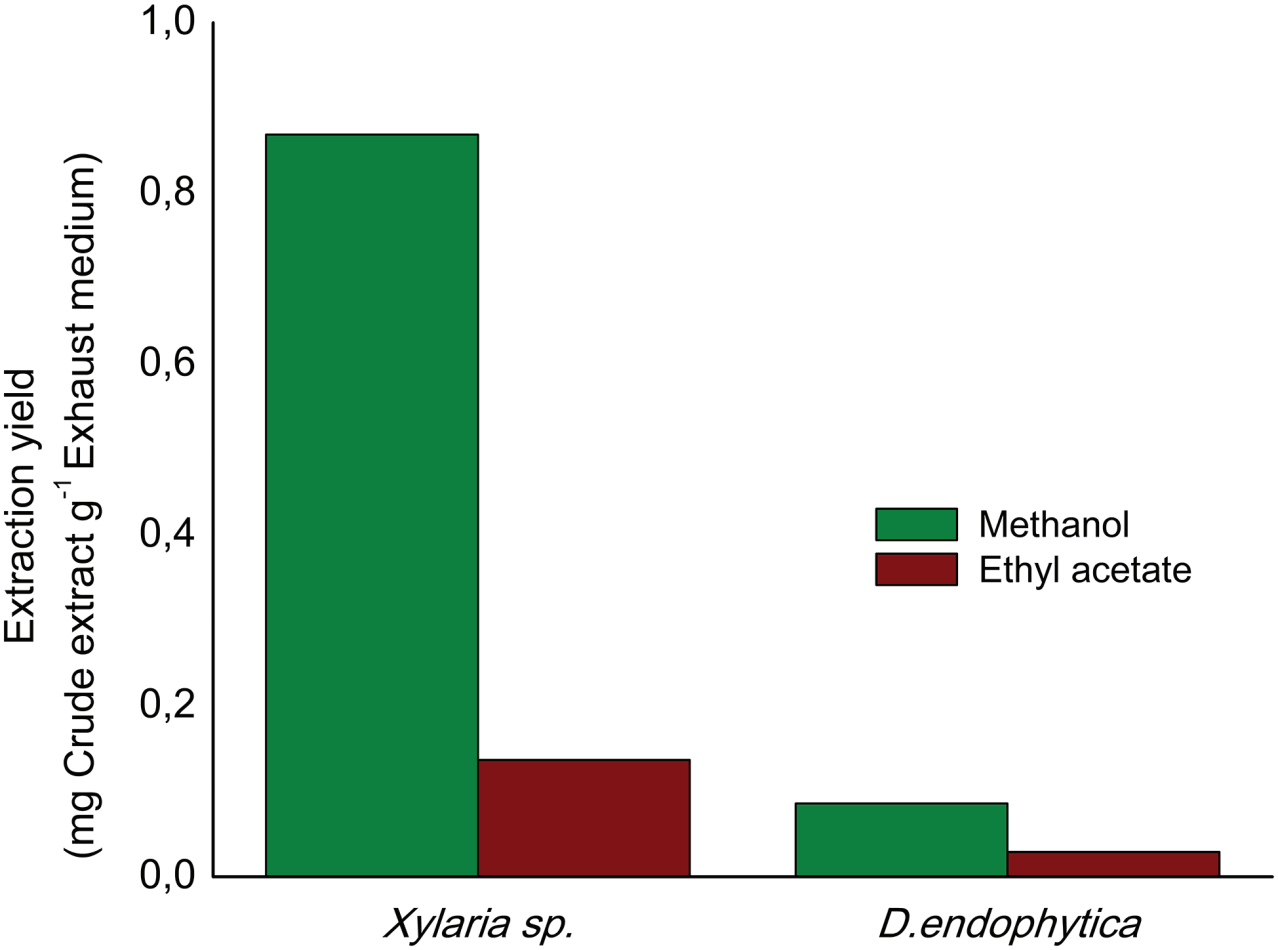
Extraction yields obtained from the alternative extraction procedure with polymeric resin Amberlite X.A.D. 16N using as eluents: ethyl acetate (EtOAc) and methanol.

The extraction yields obtained (indicated in Figure 6) with the alternative extraction procedure were lower than those obtained for both strain-extracellular crude extracts (ECEs). The elution of the resin with methanol afforded a greater than the extracted mass compared with EtOAc. However, the IC_50_ values for the methanolic fractions highlight the purification of bioactive compounds of *S. aureus*, contrary to the improved activity of the EtOAc fractions against *E. coli*. Despite this fact, the observed activity in the respective fractions for *Xylaria* sp. against *E. coli* suggested that the alternative procedure did not effectively purify the active compounds against this pathogen (Table 3). Hence, the filtrate obtained after extracting the exhausted medium with the resin was extracted with EtOAc, displaying a lower IC_50_ value against *E. coli* (1.45mg/ml).

**Table 3:** IC_50_ for fractions obtained by alternative extraction with polymeric resin Amberlite X.A.D. 16N of *Xylaria* sp. and *D. endophytica* against tested pathogens.

Strain	Eluent	IC_50_ (mg/ml)	
		*Staphylococcus aureus*	*Escherichia coli*
*Xylaria* sp.	Ethyl acetate	5.84	5.89
	Methanol	4.37	>25
*D. endophytica*	Ethyl acetate	1.83	0.99
	Methanol	0.86	9.27

### Co-culturing Screening Platform

**Figure 7:**
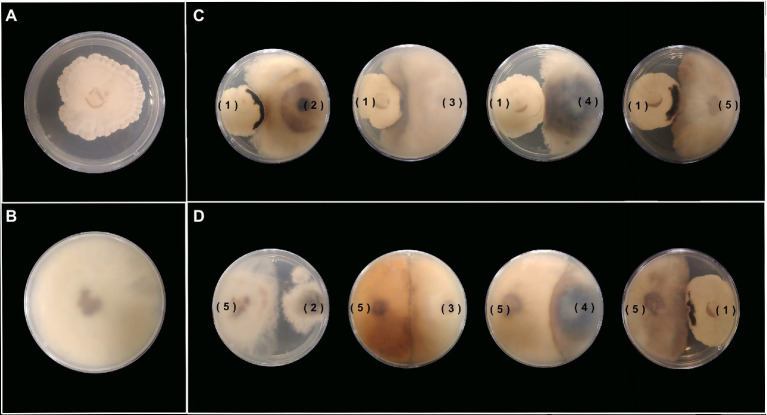
Antagonist activity between each pair of endophytic fungal strains in YM agar. **(A,B)** Anexic culture of *Xylaria* sp. (1) and *D. endophytica* (5). **(C)** *Xylaria* sp. (1) against the four strains being assessed. **(D)** *D. endophytica* (5) against the four strains being assessed.

Most of the novel compounds encountered during the past years continue to be discovered by classical screening platforms, either in the liquid culture or solid-state fermentation (Zhu et al., 2018; Becker and Stadler, 2020). However, current co-cultures have demonstrated an effective strategy to produce new secondary metabolites with various biotechnological applications. Herein, we implemented the dual culture of selected strains during the primary screening against the remaining evaluated endophytes to elucidate different fungal interactions between each pair of fungi and identify the secondary metabolite induction in contrast to the axenic cultures. Most of the assays on *Xylaria* sp. were confronted against a *Diaporthe* strain between the exhibited fungal interactions, with the development of a visible confrontation zone (zone line). In contrast, only the assay between *D. velutina* and *D. endophytica* displayed this type of interaction, as shown in Figures 7C,D. However, the dual culture between *Xylaria* sp. and *D. endophytica* as well as the dual culture of *Xylaria* sp. and *Diaporthe* sp. displayed a significant confrontation zone. The above observations suggest these models’ potential to explore the effect of this type of interaction on the generation of novel secondary metabolites and their bioactivities (Bertrand et al., 2013), contrasting with the axenic cultures (Figures 7A,B) where these responses were not recorded. Nevertheless, *Diaporthe* sp. did not exhibit any antibacterial activity during the preliminary screening, in contrast with the case of *D. endophytica*. In contrast, the dual culture of *D. endophytica* and *D. panaranensis* exhibited a contact inhibition type interaction, thereby inducing the production of red-coloured diffusible compounds that have not been generated during the axenic culture of *D. endophytica*.

**Figure 8:**
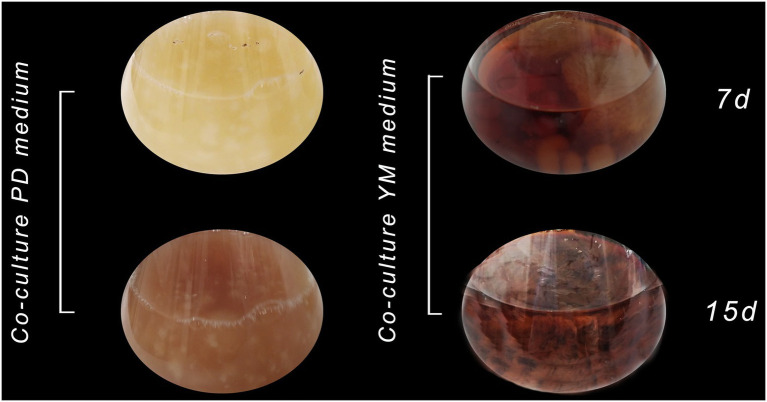
Co-culture of *Xylaria* sp. (1) and *D. endophytica* (5) in PDB and YM broth on 7 days and 15 days of incubation.

In this way, a co-culture in liquid media between *Xylaria* sp. and *D. endophytica* was performed to evaluate this strategy’s performance regarding the secondary metabolites production with antibacterial activity in different liquid media. Hence, the pre-culture time for each strain was considered in terms of the substrate consumption rate. Thus, it was decided that considering the rapid growth of *D. endophytica*, 3days for *Xylaria* sp. and 1day for *D. endophytica* was established to ensure the optimal interaction between them. After 1week, despite the culture medium, the color of the assay’s suspensions transited from a slight red to dark brown coloration (Figure 8). Interestingly, the biomass production in PDB was attenuated compared to the axenic cultures of each endophyte and the same co-culture system in YM broth. In addition, different morphotypes were recorded presumably due to the interaction between fungi in PDB, where the mycelium of *D. endophytica* could not be differentiated from the *Xylaria* sp. pellets formed in the case of YM co-culture.

Moreover, the ECEs obtained from PDB exhibited more potent inhibitory activity against both pathogens than those obtained from the axenic cultures (Table 4). However, the IC_50_ values for each pathogen were similar, which were different from the trend obtained during the preliminary screening. Moreover, the evaluation of the YM medium improved the antibacterial activity of the co-culture system, with a 3-fold reduction in the IC_50_ value for *S. aureus* in contrast to the similar value obtained for *E. coli*.

**Table 4:** IC_50_ for ECEs obtained from the co-culture of *Xylaria* sp. and *D. endophytica* in different media against the tested pathogens.

Medium	IC_50_ (mg/ml)	
	*Staphylococcus aureus*	*Escherichia coli*
PDB	1.67	1.57
YM	0.51	1.25

## Discussion

### Primary Screening

Endophytes have been proven to be a recursive source of antimicrobial compounds during the last 10years. In addition, several studies involving endophytes isolated from the medicinal plants of tropical rainforest have led to the development of novel bioactive compounds with attractive pharmaceutical applications (Martinez-Klimova et al., 2017). In this context, no research on the antibacterial potential of the endophytic community of *O. gracilipes* has been reported (Caicedo et al., 2019). In the current study, five endophytic strains of this medicinal plant corresponding to the genera *Xylaria* and *Diaporthe* were screened for their inhibitory activity against *E. coli* and *S. aureus*. The ECEs from endophytes *Xylaria* sp. and *D. endophytica* displayed broad-spectrum activity against both the tested bacteria. Simultaneously, the mycelial crude organic extracts from the most evaluated strains were bioactive only against the Gram-positive bacteria. As shown in Figure 4, most fermentation experiments produced colored compounds, indicating that the production was affected by the culture media constituents and the evaluation conditions. Thus, the use of PDB promotes the formation of colored compounds in endophytes, as reported by several other studies (Danagoudar et al., 2017; Xu et al., 2018).

On the other hand, Rao et al. (2015) studied the impact of different culture media in the antibacterial activity of *Diaporthe liquidambaris* (Synonym: *Phomopsis liquidambaris*) and suggested the use of PDB for optimal antimicrobial activity. Interestingly, Yao et al. (2016) reported such color changes during the metabolomic study of the co-culture of *T. versicolor* and *G. applanatum*, which, in their case, represented a significant increase in the metabolic bioactivity relative to that with monocultures. Among all the evaluated strains, *D. endophytica* exhibited a color transition during the fermentation time, which corresponded to its ECE’s potent bioactivity, resulting in an organic extract with the highest potential obtained during the primary screening. In addition, even when the evaluated mycelial organic crude extracts were found bioactive only against the Gram-positive pathogen, it is noting that the obtained extraction yields are within the range of obtained values for the solid fermentation of endolichenic fungi on rice media, where also Xylariaceae species presented the highest extraction yields (Santiago et al., 2021).

During the last few decades, the increase in antimicrobial resistance has urged the need for new compounds with broad-spectrum bioactivity to provide effective treatment options to MDR bacteria (Brown and Wright, 2016). The screening of antimicrobial activity using crude extracts allows the identification of potential producer strains and, in several cases, the further isolation and identification of compounds responsible for their bioactivities. However, several reports have indicated that the individual responsible molecules can have a lower activity than the crude extract containing these molecules. This behavior suggests a synergistic effect between these molecules in the respective crude extracts (Caicedo et al., 2012; Becker and Stadler, 2020). Hence, as a selection criterion during the primary screening, identifying extracts with inhibitory activity against Gram-negative and Gram-positive bacteria was applied in the present study. Therefore, the HCA results serve as the statistical proof for selecting the ECEs of *Xylaria* sp. and *D. endophytica* for follow-up studies. Moreover, these results suggest that different bioactive metabolites can be present in the mycelium extracts than the metabolites present in the ECEs, thereby displaying broad-spectrum bioactivity.

The *Diaporthe* species predominantly produce various polyketides and cytochalasins with low molecular weight, including compounds with antibacterial activity against the MDR enteropathogenic bacteria *Shigella flexneri* and *Vibrio cholerae*, as well as other MDR pathogens such as *S. aureus* and others (Jouda et al., 2016; Chepkirui and Stadler, 2017). Furthermore, the production of terpenoids and rare compounds with broad-spectrum pharmacological bioactivity has been reported from endophytic species of this genus. Dettrakul et al. (2003) obtained the diterpenes diaportheins A and B with potent antibacterial activity against Gram-negative and Gram-positive bacteria. In the case of diaportheins B, it was reported to strongly inhibit the growth of *Mycobacterium tuberculosis* (Chepkirui and Stadler, 2017). The Diaporthe endophytic species are mainly widely distributed among different ecosystems, resulting from its secondary metabolites’ chemical diversity and the capacity to interact positively with its host plant (Chepkirui and Stadler, 2017). ECEs produced by endophytes of this genus have been tested for their antibacterial activities to associate their biotechnological potential against several pathogens. In this sense, some studies have reported the antibacterial potential of diaporthe strains, with active ECEs in concentrations down to 1.25mg/ml against susceptible *S. aureus*. In contrast, for *E. coli*, a concentration >5mg/ml is required to show activity against this pathogen (De Azevedo Silva et al., 2018). Notably, in our study, the same trend was recorded, where the ECEs of diaporthe species were more active against the tested Gram-positive bacteria.

On the other side, the *Xylaria* species have provided several new compounds with diverse biotechnological applications during recent years, and most of the reported compounds were isolated from endophytic strains (Becker and Stadler, 2020). For instance, *Xylaria* can produce compounds with broad-spectrum activity, such as 7-amino-4-methyl coumarin, which displays antibacterial activity against 13 microorganisms, including *E. coli* and *S. aureus* (Liu et al., 2008). Despite this fact, crude EtOAc extracts of endophytic *Xylaria* strains have also been shown to be active against several bacteria at concentrations >1mg/ml (Hamzah et al., 2018). Even when the ECEs of *Xylaria* sp. displayed a higher IC_50_ than the reported minimum inhibitory concentration values by different authors, both are not wholly comparable. The minimum inhibitory concentration is a qualitative parameter representing the minimum concentration of the extract/compound that exhibits the inhibitory activity against a pathogen (Balouiri et al., 2016). On the other hand, the IC_50_ represents a robust parameter suitable for studying antibiotic sensitivity due to its reproducibility and statistical determination.

### Extracellular Extraction Using Polymeric Resin Adsorbent

The alternative extracellular extraction with Amberlite X.A.D 16N resulted in a decreased extraction yield, which may be explained as the only organic compound of a low molecular weight to be adsorbed from the fermentation broth. Despite the above points, the obtained fractions improved the antibacterial activity, as it allowed purifying part of the crude organic metabolites responsible for the bioactivity. In the case of the methanolic fraction of *Xylaria* sp., it exhibited more vigorous antibacterial activity against *S. aureus* than its respective ECE. Moreover, the EtOAc fraction displayed moderate antibacterial activity, suggesting that, even when this eluent allowed further purification of compounds extracted by the resin, the major compounds responsible for the observed activity against *E. coli* in the ECE were not effectively extracted with this method. Notably, the resin allows capturing compounds up to 40,000gmol^−1^, suggesting that the recovered compounds in the obtained filtrate after extracting the fermentation broth with the resin may have a higher molecular weight than those captured with the resin alternative extraction. This hypothesis was demonstrated after evaluating the bioactivity of the EtOAc extract of this remaining solution, providing an improvement in the antibacterial activity against the Gram-negative pathogen for *Xylaria* sp.

Similarly, for *D. endophytica*, the obtained fractions significantly improved the antibacterial activity against both bacteria. Specifically, the methanolic fraction provided a better purification of the active compounds against *S. aureus*. At the same time, EtOAc promoted almost a 10-fold decrease in the IC_50_ value than the observed activity of the methanolic fraction against *E. coli*.

### Co-culturing Screening Platform

Several past studies have demonstrated that co-culturing methods effectively improve the conventional screening platforms and produce unique compounds with several biotechnological applications (Bertrand et al., 2014). Herein, we developed a co-culture platform of *O. gracilipes* endophytes. For this purpose, we initially confronted the prioritized strains during a preliminary screening of antibacterial activity against the remaining evaluated fungi. Different fungal interactions were identified, but mainly pigment production was selected to indicate secondary metabolite production. Pigments produced by fungi can be attributed to different biological activities, such as antibacterial, antifungal, and herbicidal. They may be developed in response to adverse conditions or as a defence mechanism to other microbes (Hamzah et al., 2018).

In this sense, according to the significant deadlock displayed by the dual culture plate assay of *Xylaria* sp. and *D. endophytica*, we evaluated the effect of different culture media on the co-cultivation of these endophytes. We observed that when both the endophytes were cultured together in PDB, the mycelial growth was lower relative to each fungus cultured alone. This fact suggested the generation of repressing mechanisms induced by fungal interaction in this medium, but not in YM medium. Chagas et al. (2013) investigated the mixed cultivation effects on the chemical potential of endophytes isolated from the plant *Smallanthus sonchifolius*, demonstrating that, during the co-culture of *Alternaria tenuissima* with *Nigrospora sphaerica*, *A. tenuissima* produced antifungal polyketides in response to the confrontation against *N. sphaerica*. The media composition plays a significant role in the biosynthesis of secondary metabolites. In our study, two different media were evaluated for the co-culturing between *Xylaria* sp. and *D. endophytica*, where the main difference lies in the nitrogen availability. PDB appears to be a nitrogen-limited medium, which promotes a different response in terms of mycelial morphology during the co-culture compared with YM (Figure 8).

On the other hand, for both the evaluated media, the co-culture between *Xylaria* sp. and *D. endophytica* developed a dark brown coloration continuously. These results matched with a clear improvement of the IC_50_ values when compared to the values of each individually axenic culture. Several authors have reported that the bioactive compound production is enhanced with mixed cultures, which, in some cases, has been associated with the physiological responses as the production of coloured compounds during fermentation (Yao et al., 2016). In the current study, through the development of a co-culture platform, an improvement in the antibacterial activity was achieved in contrast to the displayed bioactivity during the preliminary screening of endophytic strains, providing almost a 10-fold reduction in the IC_50_ values relative to the lowest values of the ECEs obtained from axenic cultures. These findings together demonstrate that the same endophytic strains could display different biochemical profiles in response to the hosted interaction with other microorganisms, representing an opportunity to explore the biotechnological production of new natural products from native fungi.

## Conclusion

This research is the first report on the antibacterial potential of crude extracts from cultivating a fungal endophytic community isolated from the medicinal plant *O. gracilipes* in tropical mountain rain forests. The primary screening of the endophytic strains revealed the potential of *D. endophytica* and *Xylaria* sp. to produce extracellular secondary metabolites with a broad-spectrum activity against *E. coli* and *S. aureus*, which are clinical importance microorganisms. Accordingly, follow-up studies were conducted to develop a co-culture platform between the selected strains, and an alternative extraction of the bioactive extracellular compounds was performed. Thus, the experimental results demonstrated the potential of co-culture induction to promote the antibacterial activity of the produced ECEs when compared with those from the axenic culture of respective endophytes. In addition, through the alternative extraction procedure, we could elucidate the polarity nature of the metabolites responsible for the antibacterial activity of *Xylaria* sp. against *S. aureus*, improved the activity of its ECEs for this pathogen. Consequently, this study suggests the possibilities for further investigations toward developing a valuable and reproducible platform to produce novel compounds within the Colombian natural diversity, particularly for unexplored ecosystems such as the tropical rainforest.

## Data Availability Statement

The original contributions presented in the study are included in the article/supplementary material, further inquiries can be directed to the corresponding author.

## Author Contributions

EC-G, ME, AZ-M, JC, and MM performed the experiments and wrote the draft manuscript. EC-G and ME performed data analyses. NC, AD, BF, and AV-P supervised the whole work. NC and AV-P edited the manuscript. All authors contributed to the article and approved the submitted version.

## Funding

This study was supported by a grant (No: 22028) from the Ministerio de Ciencias, Tecnología e Innovación (MinCiencias), Colombia.

## Conflict of Interest

The authors declare that the research was conducted in the absence of any commercial or financial relationships that could be construed as a potential conflict of interest.

## Publisher’s Note

All claims expressed in this article are solely those of the authors and do not necessarily represent those of their affiliated organizations, or those of the publisher, the editors and the reviewers. Any product that may be evaluated in this article, or claim that may be made by its manufacturer, is not guaranteed or endorsed by the publisher.

## Abbreviations

DMSO: Dimethyl sulfoxide
EBB: Ecology-Bioprospecting-Bioprocessing
ECE: Extracellular crude extract
EtOAc: Ethyl acetate
IC_50_: Half-maximum inhibitory concentration
LB: Luria Bertani broth
MASL: Meters above sea level
MDR: Multidrug resistant
MeOH: Methanol
OD: Optical density
PDA: Potato dextrose agar
PDB: Potato dextrose broth
YM: Yeast-maltose-glucose
